# Post-stroke angiotensin II type 2 receptor activation provides long-term neuroprotection in aged rats

**DOI:** 10.1371/journal.pone.0180738

**Published:** 2017-07-03

**Authors:** Douglas M. Bennion, Jacob D. Isenberg, Allison T. Harmel, Kelly DeMars, Alex N. Dang, Chad H. Jones, Megan E. Pignataro, Justin T. Graham, U. Muscha Steckelings, Jon C. Alexander, Marcelo Febo, Eric G. Krause, Annette D. de Kloet, Eduardo Candelario-Jalil, Colin Sumners

**Affiliations:** 1 Department of Physiology and Functional Genomics and McKnight Brain Institute, College of Medicine, University of Florida, Gainesville, Florida, United States of America; 2 Department of Neuroscience and McKnight Brain Institute, College of Medicine, University of Florida, Gainesville, Florida, United States of America; 3 Department of Cardiovascular and Renal Research, University of Southern Denmark, Odense, Denmark; 4 Department of Psychiatry and McKnight Brain Institute, College of Medicine, University of Florida, Gainesville, Florida, United States of America; 5 Department of Pharmacodynamics, College of Pharmacy, University of Florida, Gainesville, Florida, United States of America; National University of Singapore, SINGAPORE

## Abstract

Activation of the angiotensin II type 2 receptor (AT2R) by administration of Compound 21 (C21), a selective AT2R agonist, induces neuroprotection in models of ischemic stroke in young adult animals. The mechanisms of this neuroprotective action are varied, and may include direct and indirect effects of AT2R activation. Our objectives were to assess the long-term protective effects of post-stroke C21 treatments in a clinically-relevant model of stroke in aged rats and to characterize the cellular localization of AT2Rs in the mouse brain of transgenic reporter mice following stroke. Intraperitoneal injections of C21 (0.03mg/kg) after ischemic stroke induced by transient monofilament middle cerebral artery occlusion resulted in protective effects that were sustained for up to at least 3-weeks post-stroke. These included improved neurological function across multiple assessments and a significant reduction in infarct volume as assessed by magnetic resonance imaging. We also found AT2R expression to be on neurons, not astrocytes or microglia, in normal female and male mouse brains. Stroke did not induce altered cellular localization of AT2R when assessed at 7 and 14 days post-stroke. These findings demonstrate that the neuroprotection previously characterized only during earlier time points using stroke models in young animals is sustained long-term in aged rats, implying even greater clinical relevance for the study of AT2R agonists for the acute treatment of ischemic stroke in human disease. Further, it appears that this sustained neuroprotection is likely due to a mix of both direct and indirect effects stemming from selective activation of AT2Rs on neurons or other cells besides astrocytes and microglia.

## Introduction

Targeting of angiotensin type 2 receptors (AT2R) in ischemic stroke by pharmacological activation has reproducibly induced decreases in infarct size and improvements in neurological function in young adult rodents when measured at early (≤ 7 days post-stroke) time points [1–9]. For example, our group was the first to report that post-stroke activation of AT2Rs with a selective agonist, Compound 21 (C21), was effective at reducing stroke-induced brain damage when administered systemically to 8–10 week old rats [4]. The mechanisms for these neuroprotective actions of AT2R in stroke appear to be multiple, and potentially include neurotrophic, anti-inflammatory, anti-chemotactic and anti-oxidant actions, as well as increasing neurogenesis, angiogenesis and cerebral blood flow [10, 11]. In addition to this, AT2R expression may be increased in ischemic brain regions after stroke [12–15] and contribute to the protective effects of AT2R agonists.

Despite these advances in demonstrating the protective role of AT2R agonists and the mechanisms involved, questions remain regarding the protective actions of AT2R in ischemic stroke. First, if AT2R agonists are to be considered as a potential novel therapeutic for ischemic stroke, it is essential to verify that the protective actions of these agents are sustained beyond the initial days after stroke. Second, it is also critical that these beneficial actions of AT2R agonists are demonstrated to occur in aged rats as more clinically-relevant model of human disease. Third, to better understand the mechanisms of action of AT2R agonists in stroke, it is important to assess the actual cellular localization of AT2R in the brain following ischemic stroke. This would help with understanding which brain cells display an increase in AT2R after stroke and which of the above-listed actions of AT2R are direct. However, progress in this area has been hampered by limitations of the available immunostaining and autoradiography techniques.

To address these points, in the present study, we have investigated the potential sustained neuroprotective effects of systemically administered C21 at reducing infarct volume and improving neurological function in aged Sprague Dawley rats using a transient middle cerebral artery occlusion (MCAO) model of ischemic stroke. We have also used a BAC-transgenic AT2R reporter mouse, an animal in which in every cell containing AT2R exhibits green fluorescence [16], to determine the cellular localization of AT2R within the cerebral infarct zone after ischemic stroke.

## Materials and methods

### Ethical approval

These experiments were approved by the Institutional Animal Care and Use Committee of the University of Florida and conducted according to the National Institutes of Health Guide for the Care and Use of Laboratory Animals, 8^th^ Ed. (2011). We have additionally adhered to the ARRIVE guidelines [17] in experiment conduct and reporting.

### Animals and housing

Rats (1 per cage) were housed in specific pathogen-free, temperature-controlled facilities (24 ± 1°C; 12–12 hour light–dark cycle) with ad libitum access to standard chow and water. Aged male Sprague-Dawley rats (n = 28, 18–20 months, 600-800g) were purchased from Hilltop Laboratories (Scottdale, PA, USA). Adult male AT2R reporter mice (n = 17, 6–8 months, 25-35g) were bred in the laboratory of Eric Krause (Department of Pharmacodynamics, University of Florida). This transgenic mouse model was generated to express green fluorescence protein (GFP) driven by AT2R regulatory sequences, with the result that all AT2R+ cells fluoresce green [16]. All animals were drug- and test- naïve prior to inclusion in this study.

### Assessments of neurological function

#### Bederson and garcia exams

Post-stroke neurological function was assessed using modified Bederson and Garcia scoring scales as we have described previously [4, 18–20].

#### Paw adhesive test

Functional sensorimotor deficits after stroke were sensitively assessed by applying a 1 cm diameter adhesive tape to the contralateral impaired paw and recording the latency to touch and removal [21]. Times that exceeded two minutes were recorded as 120 seconds. Each session included three trials, and measurements were recorded as the average of the two best trial times per session. Rats underwent two paw adhesive sessions of training before stroke followed by serial post-stroke assessments.

#### Rotarod test

Rats also underwent rotarod testing, each session of which consisted of 3–5 trials in which rotations accelerated from 4 per minute to 40 rotations per minute over several minutes. The average of the two best times per time point were normalized to the baseline scores to obtain a percentage of baseline. Rats were given two sessions of pre-training followed by serial post-stroke assessments.

### Intraluminal transient MCAO

Mice underwent transient MCAO using the intraluminal filament method as described previously [22]. This procedure was performed under isoflurane anesthesia. Through a ventral midline neck incision, the right common carotid artery (CCA), external carotid artery (ECA), and internal carotid artery (ICA) were carefully isolated. Silk suture, size 4–0, was used to permanently ligate the common carotid artery and the ECA was temporarily blocked using a vascular clip. A second silk suture was then loosely tied around the bifurcation of the CCA, followed by a small incision in the CCA approximately 2 mm proximal to the carotid bifurcation. Through this incision, a 7–0 silicone-coated filament was inserted and gently directed distally (normally 8–9 mm for mice and 18–20 mm for rats) along the ICA until a mild resistance was encountered indicating the branching of the anterior and middle cerebral artery. This occluding filament was tied in place for 45 minutes, during which time the animal could recover from anesthesia under close monitoring in a temperature controlled environment. Several minutes prior to the end of the occlusion period, animals were re-anesthetized, and the filament was removed to allow reperfusion of the MCA territory, followed by permanent ligation just proximal to the bifurcation of the ECA and the ICA from the CCA using the previously placed silk suture. Sham surgeries consisted of isolation of the CCA and permanent suture ligation without monofilament insertion.

### Experimental protocols

#### Experiment 1

To evaluate the long-term effects of post-stroke systemic administration of C21 in aged animals, rats (n = 28) were randomly assigned to receive intraperitoneal injections of sterile saline (0.9%) or C21 (0.03mg/kg) at 90 minutes, 1 day, and 2 days following intraluminal transient MCAO. The outcome measures were neurological function, assessed at 4 hours, and 1, 3, 7, 14, 21, and 28 days after stroke, and infarct volume as assessed by magnetic resonance imaging (MRI) at three-weeks post-stroke. MRI was chosen for infarct volume analyses because using TTC staining (as in our previous work [4]) was not possible in the current study, as we wanted to examine sustained behavioral protection elicited by C21 over 4 weeks after MCAO.

#### Experiment 2

Non-stroke brains from male (n = 3) and female (n = 5) AT2R reporter mice [16] were used to assess cellular localization of AT2R under control conditions. Additional male AT2R reporter mice were subjected to sham surgery or 45 minutes of intraluminal transient MCAO followed by euthanasia and tissue fixation by cardiac perfusion with saline followed by 4% paraformaldehyde (~50mL each) at 7 days post-stroke (n = 6 stroke and n = 3 sham) or 14 days post-stroke (n = 5 stroke and n = 3 sham). Brains were harvested and post-fixed in 4% paraformaldehyde overnight followed by 30% sucrose until sectioning.

### Anesthesia, analgesia, and euthanasia

Anesthesia for animal surgeries was induced using 100% O_2_/4% isoflurane and maintained using 100% O_2_/2% isoflurane. Post-operative analgesia was provided using buprenorphine (0.05 mg/kg, subcutaneously, Hospira Inc., Lake Forest, IL, USA). Euthanasia was under deep anesthesia (5% isoflurane) by decapitation for rats or cardiac perfusion for mice.

### Magnetic resonance imaging scans and image analysis

At three weeks after stroke induced by intraluminal transient MCAO, aged rats from were taken to the University of Florida’s Advanced Magnetic Resonance Imaging and Spectroscopy Facility (AMRIS) for MRI using methods developed by Dr. Marcelo Febo (University of Florida, Department of Psychiatry) and described here. During imaging, rats were anesthetized under 1.5% isoflurane gas, placed in a body tube cradle, and setup in a 96 surface transmit/receive radio frequency coil system used for high-resolution imaging on a Magnex Scientific 4.7 Tesla MR scanner. T2 relaxometry pulse sequences were run on a VnmrJ 3.1 console (Agilent, Palo Alto, CA, USA). Core body temperature and respiratory rates were monitored throughout the experiments. For T2 relaxation, the following parameters were used: echo time (TE) = 37.5 ms, repetition time (TR) = 2000 ms, target b value = 1269.92 s/mm^2^, field of view 25.6 mm^2^ along the read and phase directions and 1.5 mm along the slice direction, and data matrix of 96 x 96 x 8 brain slices. Signal averaging was used to increase signal to noise. Images were imported to ImageJ (NIH) for processing of T2 maps. T2 maps were reconstructed from a log linear regression of the multi-TE value datasets. Infarct volume was measured by an investigator blinded to treatment conditions. Infarct area was calculated for each of the eight, consecutive coronal MRI brain slices, and the total volume of infarct for each slice was calculated by multiplying the infarcted area by the thickness of the slice (1mm). It was clear that the most rostral brain slice (# 1) and the two most caudal brain slices (#’s 7 and 8) displayed either no or minimal infarct in both the control and C21-treated groups, and so were excluded from the analysis. Thus, *total* hemispheric infarct volume was assessed by averaging data from MRI brain slices 2, 3, 4, 5 and 6 in both the control and C21-treated groups; slices 2, 3 and 4 are equivalent to the brain area analyzed by TTC staining in our previous studies on C21 [4]. In T2 images, the area of infarct appears lighter than healthy tissue due to the extra free water content from edema.

### Inclusion and exclusion criteria

Among the 28 aged rats that underwent intraluminal transient MCAO, five died in the initial 48-hours post-stroke (4 from control and 1 from C21 group), and an additional five were excluded (3 from control and 2 from C21 group) based on having met both the following exclusion criteria: 1) No or very limited neurological deficits by behavioral assessments at one day post-stroke; 2) no evidence of infarct on magnetic resonance imaging at three-weeks post-stroke. Mouse surgeries were without mortality or exclusion.

### Randomization and allocation concealment

Animals were identified by an assigned number and randomized using the randomize function in Microsoft Excel. Neurological assessments and analyses of all samples were performed by investigators blinded to group allocation.

### Immunohistochemistry and micrograph imaging

GFP immunoreactivity (measured as a direct index of AT2R expression) and its co-localization with neurons, microglia, or astrocytes was assessed using 30μm brain sections obtained from AT2R reporter mice brains fixed as described above in Experimental Protocols *Experiment 2*. Sections were primary labeled with mouse anti-HuC/D (human neuronal protein, 1:500) or mouse anti-NeuN (neuronal nuclear antigen, 1:500), rabbit anti-Iba-1 (ionized calcium binding adaptor molecule 1, 1:1000), rabbit anti-GFAP (glial fibrillary acidic protein, 1:1000), or chicken anti-GFP (green fluorescence protein, 1:500), and secondary labeled with Alexa Fluor® donkey anti-mouse 488 or donkey anti-rabbit Cy3 before mounting in polyvinyl alcohol or Vectashield with DAPI (4',6-diamidino-2-phenylindole) followed by fluorescence imaging using either an Olympus BX41 fluorescence microscope or a Zeiss AxioImager fluorescent Apotome Microscope with Axiovision 4.8.2 software.

### Chemicals

Chicken anti-GFP and mouse anti-HuC/D were purchased from ThermoFisher Scientific Life Technologies (Grand Island, NY), mouse anti-NeuN from Millipore (Bedford, MA, USA), rabbit anti-Iba-1 from Wako (Osaka, Japan), and rabbit anti-GFAP from Sigma-Aldrich (St. Louis, MO). (Alexa Fluor® donkey anti-rabbit 594 and anti-mouse 488 were from Molecular Probes, Invitrogen (Carlsbad, CA, USA). Alexa Fluor® donkey anti-rabbit Cy3, donkey anti-mouse Cy3, and donkey anti-chicken 488 were from Jackson ImmunoResearch Laboratories, Inc. (West Grove, PA, USA). Vectashield mounting medium with DAPI was from Vector Laboratories (Burlingame, CA, USA). All other chemicals were purchased from Fisher Scientific (Pittsburgh, PA, USA).

### Compound 21

Compound 21 (C21; MW 497.6) is a selective agonist for the AT2R (Ki of ~0.4 nM for the AT2R, and >10 μM for the angiotensin type 1 receptor) [23]. C21 was prepared freshly for each experiment. It was dissolved in 0.9% saline at a concentration of 1.0 mg/ml, and was then diluted in the same solvent to a concentration of 0.03 mg/ml. It was stored on ice until ready for use. C21 was injected intraperitoneally at a dose of 0.03 mg/kg, i.e. a 600g rat was injected with 0.6 ml of 0.03mg/ml C21 solution.

### Data analyses

Data are expressed as means ± SEM. Sample size calculations assumed a standard deviation of 15%, an effect size of 15%, power of 0.8, and alpha < 0.05. Statistical significance was evaluated with the use the Mann-Whitney test.

## Results and discussion

### Sustained neuroprotection by post-stroke AT2R agonist C21 in aged rats

**Fig 1:**
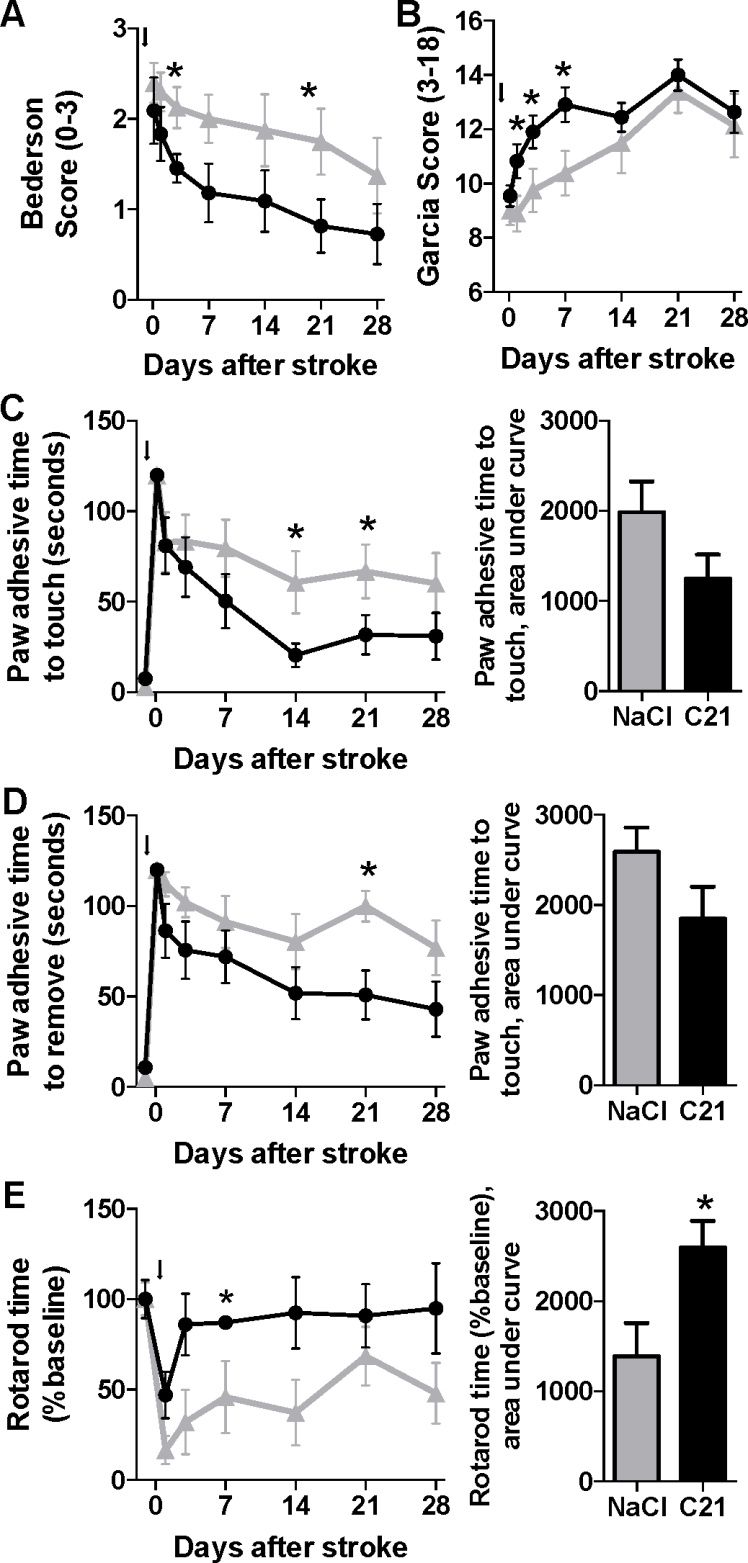
Post-stroke intraperitoneal-injected C21 results in sustained improvements in neurological function in aged rats. Behavioral tests were performed at the time points indicated after stroke (black arrow) in rats treated with intraperitoneal injections of saline (n = 8, grey triangles) or C21 (0.03mg/kg, n = 11, black circles), and included the Bederson exam (**A**), Garcia exam (**B**), paw adhesive time to touch (**C**) or removal (**D**) of paw adhesive, and rotarod performance time (**E**). Note: n = 3 saline and 4 C21-treated rats for the rotarod test. Data are mean ± SEM; *p<0.05 versus control for respective time points by Mann-Whitney test.

To assess the long-term efficacy of post-stroke injections of C21in aged rats, experiments were performed as described in *Experiment 1* of the Experimental Protocols section of the Methods. This experiment was designed to test whether AT2R-dependent neuroprotection that has been reported in young animals during the initial days after stroke could be durably induced in a model that more closely approximates the population of aged stroke patients. Intraperitoneal injections of C21 (0.03mg/kg) starting at reperfusion after 90 minutes of intraluminal transient MCAO, resulted in sustained improvement in neurological function as assessed by Bederson and Garcia exams (Fig 1A and 1B), paw adhesive testing (Fig 1C and 1D), and rotarod performance (Fig 1E), beginning in the first 24 hours after stroke and persisting through at least 3 weeks post-stroke.

**Fig 2:**
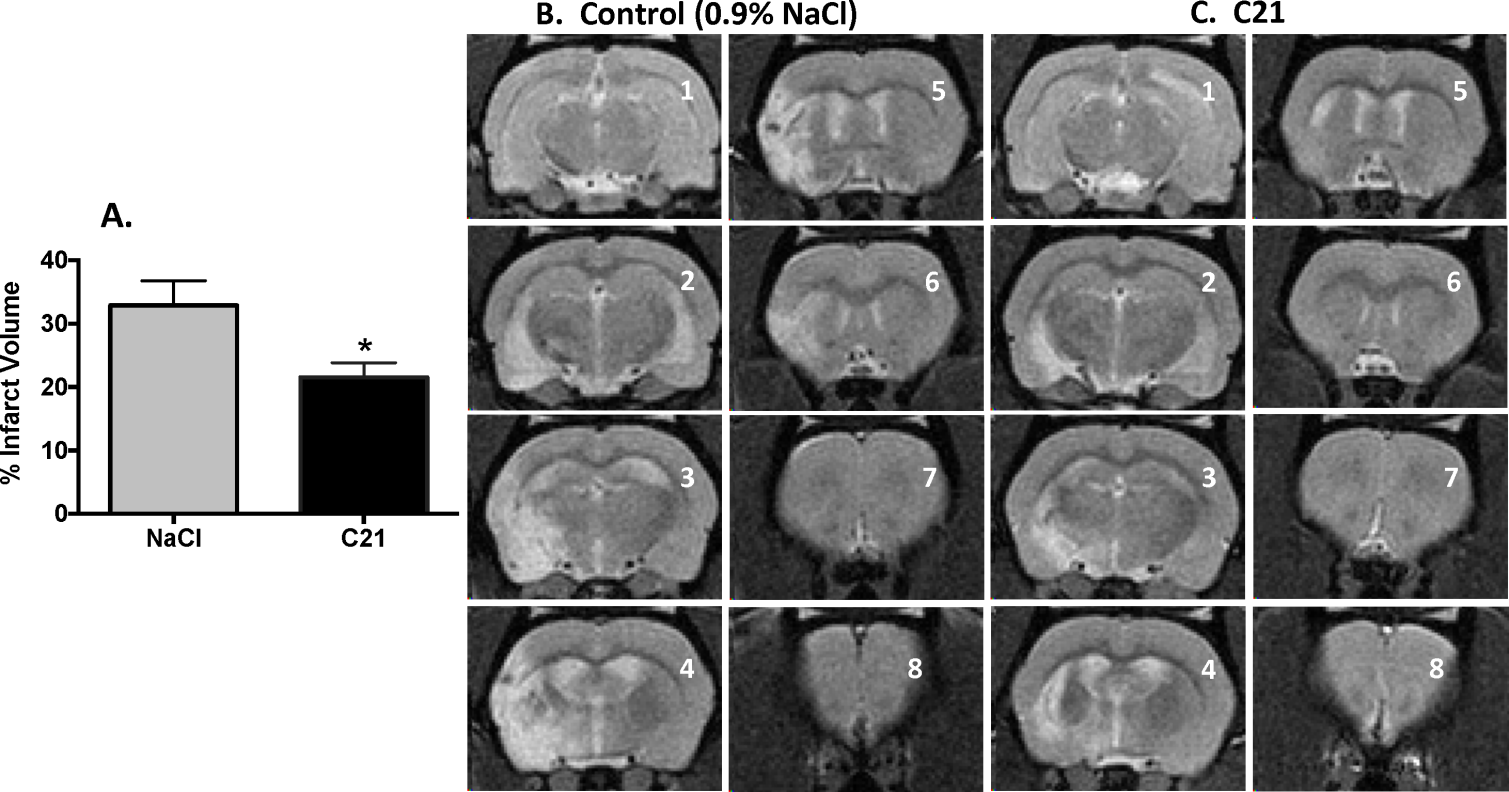
Post-stroke intraperitoneal injections of C21 result in long-term reductions in infarct volume in aged rats. Magnetic resonance images (MRIs) were obtained three weeks after induction of ischemic stroke by 90-minute monofilament occlusion of the middle cerebral artery followed by post-stroke treatment with control solution (0.9% saline; n = 7) or C21 (0.03mg/kg, n = 10). Infarct volume was computed as described in the Methods. (**A**) Bar graphs are the composite infarct volume for MRI slices 2, 3, 4, 5 and 6 under each treatment condition. Data are mean ± SEM; *p = 0.019 as compared to respective control slices. Also shown are eight representative MRI slices (1 = rostral; 8 = caudal) slices from animals treated with either 0.9% saline (**B**) or C21 (**C**).

Analysis of *total* hemispheric infarct volume, as assessed by volumetric analysis of magnetic resonance images of MRI brain slices 2, 3, 4, 5 and 6 at 3-weeks post-stroke, demonstrated that C21-treatment elicited a significant (p = 0.019) decrease in cerebral infarct volume (Fig 2A–2C). Shown in Fig 2B and 2C are representative MRI images taken from 0.9% saline and C21-treated rats, illustrating a greater level of edematous tissue (white area) within the slices from the saline group.

The AT2R is a promising new target for neuroprotective treatments in ischemic stroke. We demonstrated in these experiments that among aged rats, post-stroke administration of C21, an AT2R agonist, results in sustained significant reductions in infarct volume and improvements in neurological function. It is also noteworthy that of the five aged rats that died during the initial 48-hours post-stroke, 4 were from the control group, with only 1 from the C21 group, a further indication of the protective action of this AT2R agonist. These data represent an important step on the pathway to translating this experimental treatment toward clinical application. The Stroke Treatment Academic Industry Roundtable (STAIR) criteria recommend that preclinical testing of neuroprotective compounds include experiments in aged animals, be replicated in multiple stroke models, and assess multiple outcome measures over a sustained period of weeks, among other criteria [24]. The design of our study addresses several of these criteria for the first time regarding compounds targeting AT2Rs in preclinical studies. Until now, studies were performed in young adult rats, both healthy [4, 7, 9] and hypertensive [1, 3, 6], and in mice [2, 5, 8] with assessments typically taken out to only several days post-stroke. Here, we found that the neuroprotective effects observed in these studies in young animals were reproduced in aged rats (18–20 months), which are also highly likely to suffer from metabolic syndrome and related disorders (unpublished assessments), and that the benefits of C21 treatment were sustained for at least 3 weeks after stroke (Figs 1 and 2).

Our analysis was complicated by the unexpectedly high variability in infarct volumes among the control animals in this study, which may have resulted at least in part from differing levels of resolution of the stroke-induced edema as measured by MRI at 21-days post MCAO. We suspect that the repair mechanisms activated in the aged rat brain lead to a highly variable degree of recovery of the cerebral vasculature and accompanying edema that may not fully reflect the degree of neuronal loss and recovery that is evidenced by the functional data (Fig 1). It is also possible that variability in recovery is greater in the control rats versus the C21 treated rats, where protection has occurred. Addition of an earlier measurement of infarct volume (e.g. 1 week) could likely have provided more clarity on this question, but the fragile health of these aged animals has been found in our hands to prohibit the use of prolonged general anesthesia too close to the time of stroke surgery. Having now been reproducibly demonstrated in a broad variety of relevant preclinical conditions to induce neuroprotective effects, this protective axis of the renin angiotensin system stands as an especially promising target for clinical testing toward a new and efficacious treatment for ischemic stroke.

### Effect of stroke on distribution and cellular localization of brain AT2Rs in reporter mice

**Fig 3:**
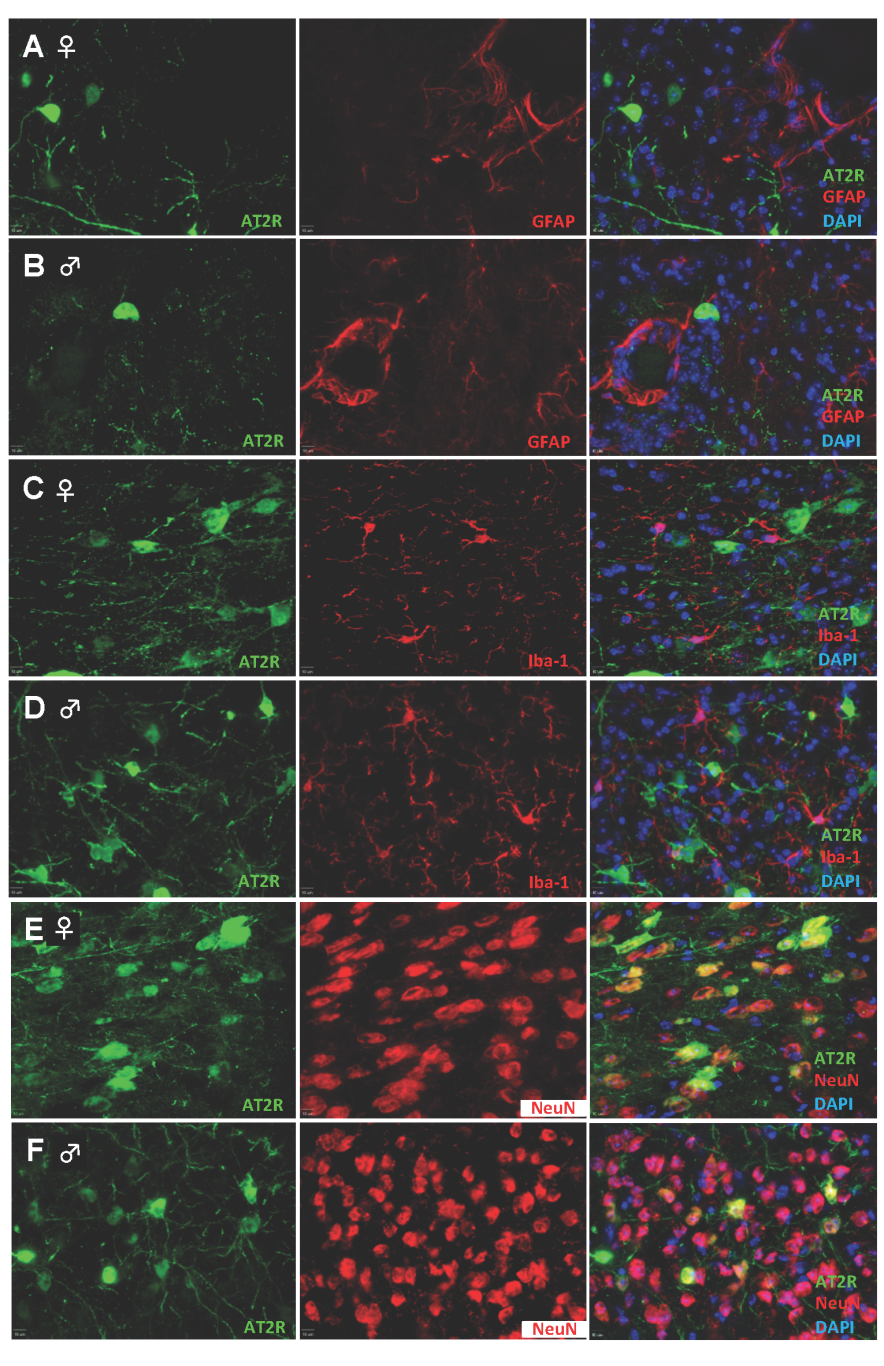
Cellular location of AT2R within the cerebral cortex is not different between female and male AT2R reporter mice. Representative high power micrographs from cerebral cortical brain regions of naïve female (**A**, **C, and E**) or male (**B**, **D, and F**) AT2R reporter mice with staining for cells immunopositive for GFP-AT2R (green, left column), GFAP (red, rows A and B), Iba-1 (red, rows C and D), or NeuN (red, rows E and F), along with DAPI co-staining (blue, right column). AT2R: angiotensin II type 2 receptor; GFAP, glial fibrillary acidic protein; GFP: green fluorescence protein; Iba-1: ionized calcium-binding adapter molecule 1; NeuN: neuronal nuclear antigen.

To characterize the cellular location of AT2R, we imaged brain sections from middle-aged (6–8 month old) AT2R reporter mice as described in *Experiment 2* of the Experimental Protocols section of the Methods. Immunostaining of coronal brain sections from non-stroke AT2R reporter mice revealed that the cellular location of AT2R in the cerebral cortex did not differ between female and male mice (Fig 3). Specifically, in mice from both genders, AT2R were localized to NeuN–positive cells (neurons, Fig 3E and 3F), but were absent from GFAP-positive cells (astrocytes, Fig 3A and 3B) and Iba-1 positive cells (microglia, Fig 3C and 3D).

**Fig 4:**
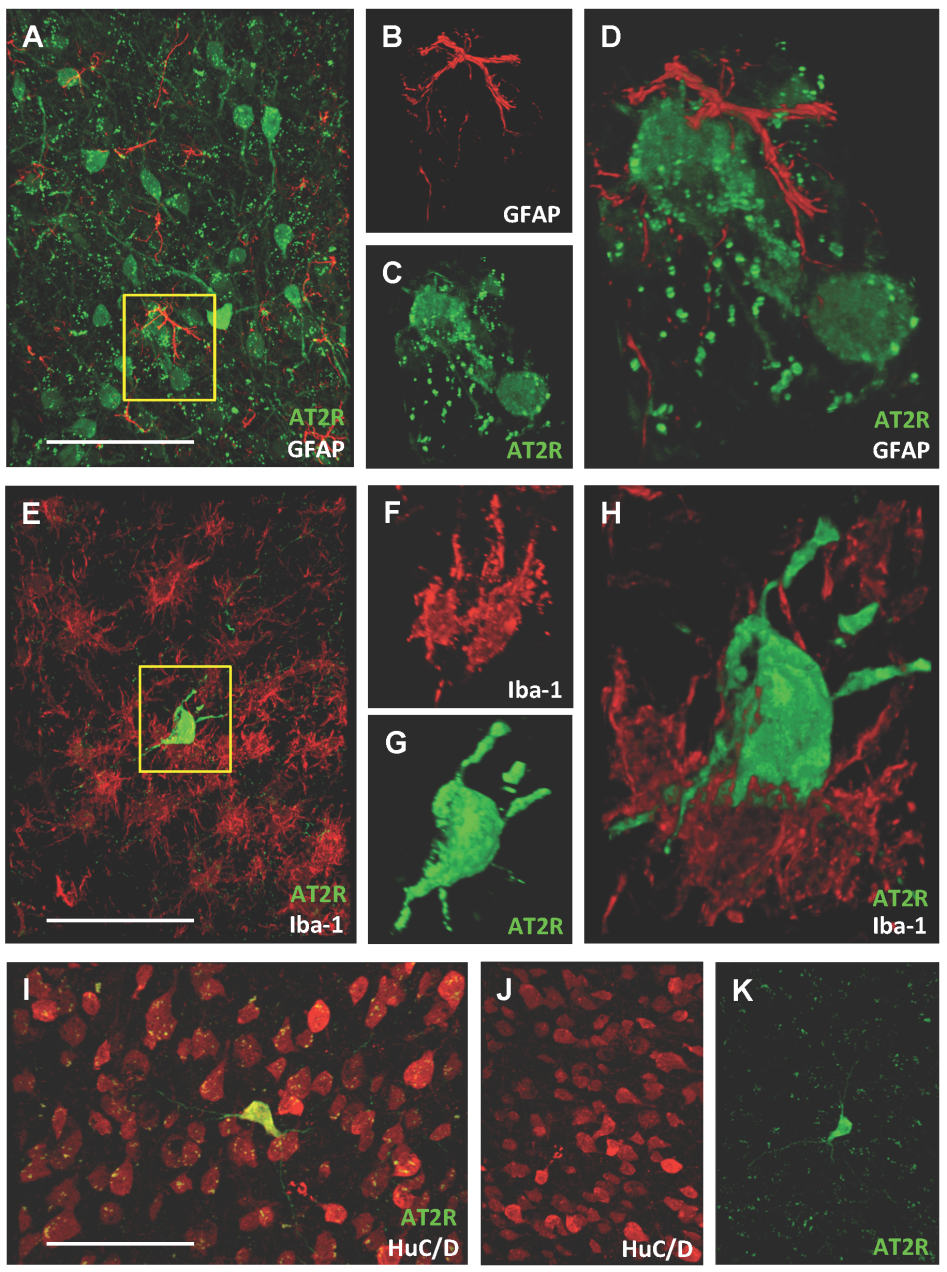
Ischemic stroke does not change the cellular localization of AT2R, which remain stably expressed on neurons. Representative high power micrographs of GFAP (red) or GFP-AT2R (green) immunopositive cells (**A-D**) or of Iba-1 (red) or GFP-AT2R (green) immunopositive cells (**E-H**). High power micrographs of HuC/D (red) and GFP-AT2R (green) immunopositive cells (**I-K**). Areas of higher magnification (b, c and f, g) are identified by the yellow inset in panels a and e, respectively. AT2R: angiotensin II type 2 receptor; GFAP, glial fibrillary acidic protein; GFP: green fluorescence protein; HuC/D: human neuronal protein; Iba-1: ionized calcium-binding adapter molecule 1. Scale bars in panels A, E and I are 50 μm.

To determine whether glia express AT2R following ischemic stroke, we performed intraluminal transient MCAO in AT2R reporter mice followed by assessments of AT2R co-localization with astrocytes (GFAP), microglia (Iba-1), and neurons (HuC/D). We did not find any co-localization of AT2R with the astrocytic marker GFAP (Fig 4A–4D) or the microglial marker Iba-1 (Fig 4E–4H) in any brain regions either ipsilateral or contralateral to the stroke at 7- or 14-days post-insult. Higher power micrographs showed no overlap of staining even in instances of close proximity of GFAP+ (Fig 4D) or Iba-1+ (Fig 4H) cells to the AT2R+ cells. By contrast, co-localization with neuronal marker HuC/D was clear in these AT2R+ cells (Fig 4I).

There are few studies of AT2R expression, in part due to limitations of available techniques to localize this receptor. The available data indicate a time course for early changes in AT2R expression following stroke. Previous studies in rats found increased AT2R expression in the ipsilateral hemisphere at 24 hours following cerebral ischemia [12]. A later study found that AT2R expression and distribution at 2-days post-stroke were increased as compared to brains from sham operated rats [13]. Most recently, it was reported that the number of AT2R+ cells in ischemic regions increased to a peak level at 3 days post-intraluminal MCAO and decreased by 7 days toward sham levels [15]. Additionally, this study reported co-localization of AT2Rs with neuron markers NeuN, a finding that is in line with our observations (Figs 3 and 4). The absence of a change in the cell-subtype AT2R expression does not disrupt the consensus from earlier studies that describes an early time course of AT2R expressional changes after stroke, since our evaluation was performed as a non-quantitative assessment and at later time points, 7 days and then again at 14 days. As such, it is possible that there is early up-regulation of AT2Rs in the initial hours and days within neurons and possibly other cell types following stroke with subsequent down-regulation in non-neuronal cells.

We found the AT2R to be localized to neurons (Figs 3E, 3F and 4I–4K), where its activation by AT2R agonists in ischemic stroke models has been shown to reproducibly reduce markers of apoptosis or increase neuron survival in vitro [1, 2] and in vivo [6, 7]. Studies have also demonstrated a robust anti-oxidative effect of AT2R activation in ischemic brain tissue [1, 4, 5, 7], with treatments resulting in lower levels of nitric oxide synthase enzymes and superoxide and nitrative free radicals that are damaging to neurons. Activation of AT2Rs appears also to be linked to the production/release of neurotropic factors such as BDNF [6, 7] and the anti-inflammatory chemokine IL-10 [7], while simultaneously decreasing the expression of TNF-α and certain potentially deleterious chemoattractant factors, such as monocyte chemoattractant protein-1 (also known as C-C chemokine ligand 2) and C-C chemokine receptor type 2 [4, 5] that could induce excessive inflammatory cell migration and activation in areas of vulnerable tissue.

Regarding glial cells, AT2R activation after stroke induction does not appear to have a profound effect on the activation status of astrocytes and microglia/monocytes since several studies showed no significant effects on markers of their activation [4, 6, 7]. This is in line with our observation that astrocytes and microglia do not express AT2Rs at baseline (Fig 3A–3D) or after stroke (Fig 4A–4H), and thus cannot be directly modulated by AT2R agonists. One study found an increased number of activated microglia in response to pre- and post-stroke treatments with an AT2R agonist, but not post-stroke only treatments [6]. An earlier study from this group also showed that post-stroke treatments with the peptide AT2R agonist CGP42112 led to increased activation of microglia in the core, but not penumbra [3]. Activation of AT2Rs on neurons may reduce neuroinflammation in and around this area through indirect neuronal AT2R-driven mechanisms, such as by C21-induced decreases in C-C chemokine receptor and ligand expression noted above, which may limit invasion into damaged areas of inflammatory cells. We also note that due to the relatively limited distribution of brain AT2Rs to neurons only, another possible explanation for the protective action of C21 is the non-selective activation of the Mas receptor, especially considering the potential for inhibition of C21-induced effects by Mas receptor antagonist A-779 [25, 26].

Our analysis does not preclude an important role for vascular cells, such as endothelial cells, pericytes, and smooth muscle cells, in the protection afforded by AT2R signaling via effects on both perfusion and angiogenesis. AT2R activation has been found to increase cerebral blood flow during periods of reperfusion [2] and of recovery in the days following ischemia [5], although data from our group did not uncover an acute effect of AT2R activation by intraperitoneal injection of C21 on cerebral blood flow under conditions of baseline or during endothelin-1 induced MCAO [4]. At high enough doses, C21 has been shown to induce vasorelaxation in isolated rat basilar arteries [6]. Treatment with C21 initiated at the time of reperfusion in mice has also found to decrease the permeability of the BBB [5], although the mechanisms remain unclear. AT2R activation by C21 in the hours after stroke may also result in increased vessel growth in ischemic penumbral areas and subsequent tissue salvage, since C21 increases the number of vascular profiles in the rat brain by treatment and that the migration of human cerebromicrovascular endothelial cells in a BDNF-dependent manner [7]. VEGF expression in stroke is also persistently higher following treatments with C21 [9]. It remains to be clarified whether these vasoprotective effects of AT2R activation are due to indirect signaling mediated by neuronal AT2R activation or due to direct signaling by AT2Rs that may be expressed on vascular cells.

In summary, the sustained neuroprotection in ischemic stroke that we and others have observed is likely driven directly by neuronal or possibly vascular AT2R signaling in combination with indirect effects on glial and inflammatory cells. Having now been reproducibly demonstrated in a broad variety of relevant preclinical conditions to induce neuroprotective effects, this protective axis of the renin angiotensin system stands as an especially promising target for clinical testing toward a new and efficacious treatment for ischemic stroke.
